# LpMAX2 Is a Strigolactone/Karrikin Signaling Component in Perennial Ryegrass (*Lolium perenne* L.)

**DOI:** 10.3390/ijms27010031

**Published:** 2025-12-19

**Authors:** Haiyang Yu, Fang Qiu, Yuehua Wang, Ruifeng Yao, Meng Zhang, Li Chen

**Affiliations:** 1 State Key Laboratory of Chemo and Biosensing, Hunan Provincial Key Laboratory of Plant Functional Genomics and Developmental Regulation, College of Biology, Longping Agricultural College, Hunan University, Changsha 410082, China; yuhaiyang@hnu.edu.cn (H.Y.); qiufangyanling@163.com (F.Q.); wyhua@hnu.edu.cn (Y.W.); ryao@hnu.edu.cn (R.Y.); 2 Hunan Research Center of the Basic Discipline for Cell Signaling, College of Biology, Hunan University, Changsha 410082, China; 3 Yuelushan Laboratory, Changsha 410128, China

**Keywords:** *Lolium perenne*, LpMAX2, drought stress, strigolactone/karrikin signaling pathway, *Arabidopsis thaliana*

## Abstract

Perennial ryegrass is a widely cultivated cool-season forage and turf grass species whose growth and development are limited by drought and high temperature. MAX2 is an F-box leucine-rich repeat (LRR) protein, which serves as a central component of strigolactone (SL) and karrikin (KAR) signaling pathways, involved in multiple growth and developmental processes as well as stress response. Here, we identified LpMAX2, a perennial ryegrass (*Lolium perenne* L.) homolog of *Arabidopsis* MAX2 (AtMAX2) and rice D3. LpMAX2 can interact with AtD14 and LpD14 in an SL-dependent manner, implying functional conservation with AtMAX2. Overexpression of *LpMAX2* in the *Arabidopsis max2-3* mutant partially rescued leaf morphology, hypocotyl elongation, and branching phenotypes, while fully restoring drought tolerance, highlighting the evolutionarily conserved roles of MAX2 in plant growth and drought resistance. In conclusion, LpMAX2 is evolutionarily conserved in SL/KAR signaling pathways, highlighting its potential function in drought adaptation. In addition to elucidating the biological function of LpMAX2, this study identifies a promising genetic target for enhancing stress resilience in forage grasses through biotechnological approaches.

## 1. Introduction

Perennial ryegrass (*Lolium perenne* L.) is a widely cultivated cool-season perennial grass species extensively cultivated for its dual utility as high-quality forage and durable turf grass. However, its productivity is increasingly threatened by drought stress, which has become more severe due to climate change. Global analyses reveal that persistent multiyear drought has expanded terrestrial areas at a rate of 49,279 ± 14,771 km^2^/year from 1980 to 2018 [1], substantially reducing grassland ecosystem productivity worldwide [2].

Drought is one of the most significant abiotic stresses affecting global agriculture, profoundly impacting plant growth and developmental processes. Through evolutionary adaptation, plants have developed intricate physiological and molecular mechanisms to acclimate to both transient and prolonged drought conditions [3,4]. Plant hormone signaling is crucial for adaptation and tolerance to environmental stress, including abiotic stresses, such as drought, salinity, heat, cold, flooding [5], and biotic stresses, such as pathogen infections, insect pests, and herbivory [6]. The molecular mechanisms through which classical plant hormones mediate drought stress responses have been systematically investigated and comprehensively elucidated. For example, Abscisic acid (ABA) is considered to be the main hormone which intensifies drought tolerance in plants through various morpho-physiological and molecular processes, including stomata regulation, root development, and initiation of the ABA-dependent pathway [7]. In addition, jasmonic acid (JA), salicylic acid (SA), ethylene (ET), auxins (IAA), gibberellins (GAs), cytokinins (CKs), and brassinosteroids (BRs) are also very important phytohormones to congregate the challenges of drought stress [8]. As novel plant hormones, strigolactones (SLs) are a class of bioactive sesquiterpene lactones which are derived from carotenoids that inhibiting plant shoot branching [9,10], regulating root system architecture [11], symbiotic rhizosphere signals [12], and also regulating abiotic stress, such as drought and salt stress via many stress- and/or ABA-responsive genes and cytokinin metabolism-related genes [13,14].

The core SL signaling pathway in *Arabidopsis thaliana* and rice involves a protein complex comprising DWARF14 (D14), the F-box protein MORE AXILLARY GROWTH 2 (MAX2), and SUPPRESSOR OF MAX2-LIKE 6/7/8 (SMXL6/7/8) proteins [15,16,17,18]. Notably, MAX2 serves as a key regulatory node, participating in both SL signaling (it was recruited by D14) and karrikin (KAR) pathways (it was recruited by D14L/KAI2) [19]. The *Arabidopsis max2* mutant exhibits hypersensitivity to drought stress, characterized by increased water loss compared to wild-type (WT) plants. This may possibly be attributed to a thinner cuticle and impaired stomatal closure due to reduced ABA sensitivity [20], and defective SL-mediated stomatal regulation, as observed in both *max2* and *d14* mutants [21]. *Sapium sebiferum SsMAX2* overexpression (OE) in *Arabidopsis* significantly promoted resistance to drought, osmotic, and salt stresses [22]; and *Malus domestica MdMAX2* overexpressed apple calli, and in *Arabidopsis*, it exhibited increased tolerance to salt and drought stresses [23]; and Cucumber (*Cucumis sativus*) *CsMAX2* overexpression in *Arabidopsis* significantly promoted resistance to drought, ABA, and salt stresses [24]. WGCNA analysis correlating transcriptome data revealed that the hub genes D3 (MAX2) and HOX12 regulate tiller to adapt to environmental stress under Repeated Drought–Rewatering Cycles in perennial ryegrass [25]. In addition, the MAX2–KAI2–SMAX1 complex, with the assistance SA signaling to fine-tune immune responses [26], and NaMAX2 function in high-light adaptation and fitness optimization by regulating high-light responses independently of its roles in the SL and KAR signaling pathways in *Nicotiana attenuate* [27]. MAX2 ubiquitinates WRKY41, thus marking it for cold-induced degradation and thereby alleviating the repression of CBF expression to adapt to freezing [28]. These findings collectively demonstrate that MAX2 can be one of the main regulators of plant stress adaptation, integrating multiple signaling pathways to optimize responses to abiotic and biotic challenges. Although the role of the F-box protein MAX2 in drought tolerance has been studied in various plant species, its function in perennial ryegrass (*Lolium perenne*) remains uncharacterized.

In this study, a perennial ryegrass F-box protein LpMAX2 was identified. LpMAX2 contains a highly conserved F-box domain. Subcellular localization confirmed its nuclear presence, consistent with its *Arabidopsis* ortholog AtMAX2. The overexpression of *LpMAX2* in an *Arabidopsis max2-3* mutant partially complemented several phenotypic defects, including leaf shape, hypocotyl elongation, and primary branching. In addition, the overexpression lines enhanced drought tolerance, accompanied by the upregulation of drought-responsive markers (*RD22*, *RD29A*, and *RAB18*), indicating the conserved role of LpMAX2 in plant drought resistance. These findings are from the first systematic functional analysis of LpMAX2 in drought resistance in perennial ryegrass. This study offers a prime genetic target for improving ryegrass drought tolerance via molecular breeding or genetic engineering.

## 2. Results

### 2.1. Identification and Sequence Analysis of LpMAX2 in Perennial Ryegrass

**Figure 1:**
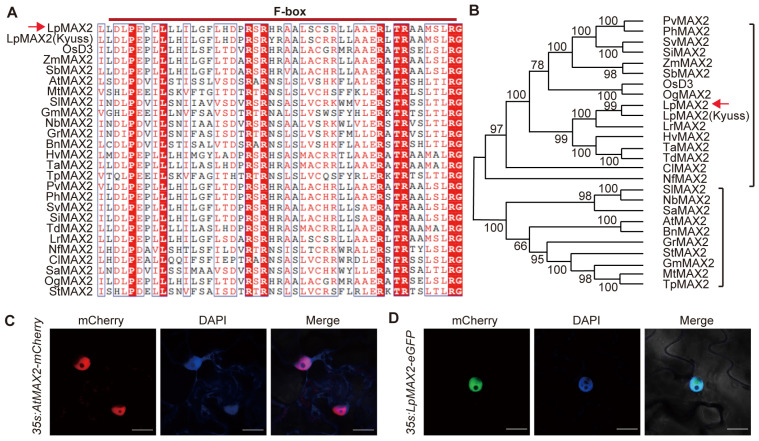
Characterization and localization of LpMAX2. (**A**) The figure shows a fragment of the amino acid sequence alignment of LpMAX2 (XP_051197565.1) with MAX2 proteins from multiple species including *Oryza sativa* (XP_025882032.1), *Zea mays* (XP_020394883.1), *Sorghum bicolor* (XP_002436499.1), *Arabidopsis thaliana* (NP_565979.1), *Medicago truncatula* (XP_003607592.1), *Solanum lycopersicum* (XP_004243284.1), *Glycine max* (XP_003540983.1), *Nicotiana tabacum* (XP_016475919.1), *Gossypium raimondii* (XP_012437380.1), *Brassica napus* L. (XP_048634693.1), *Hordeum vulgare* (XP_044957656.1), *Triticum aestivum* (XP_044431310.1), *Trifolium pratense* (XP_045812288.1), *Panicum virgatum* (XP_039842470.1), *Panicum hallii* (XP_025812611.1), *Setaria viridis* (XP_034590675.1), *Setaria italica* (XP_004964817.1), *Triticum dicoccum* (XP_037465493.1), *Lolium rigidum* (XP_047075323.1), *Nervilia fordii* (WBO25877.1), *Carex littledalei* (KAF3341515.1), *Striga asiatica* (GER56460.1), *Oryza glaberrima* (XP_052158925.1) and *Senna tora* (KAF7833919.1); the red arrows mark LpMAX2. Red lines on the top indicate F-box domains. (**B**) Phylogenetic analysis of MAX2 proteins shown in (**A**); the red arrow marks LpMAX2. (**C**,**D**) The localization of AtMAX2 (**C**) and LpMAX2 (**D**) protein in leaf cells of *Nicotiana benthamiana*. *1300-eGFP-AtMAX2* and *1300-eGFP-LpMAX2* plasmids were transformed into leaf cells of *Nicotiana benthamiana* via agrobacterium-mediated transformation. GFP fluorescence was visualized using confocal microscope. Nuclei are indicated by DAPI staining. Bars = 100 µm.

The protein sequence of OsMAX2(D3) (XP_025882032.1) served as a query for BLASTP searches against the NCBI non-redundant database https://www.ncbi.nlm.nih.gov/ (accessed on 10 April 2022) to identify MAX2 homologs in closely related species. A 2178 bp sequence encoding a 726-amino acid polypeptide was isolated using PCR assay from a cDNA library of perennial ryegrass (*Lolium perenne*). This sequence exhibits 97.9% similarity with the published MAX2 gene sequence of the perennial ryegrass cv. Kyuss. Therefore, this gene was designated as *LpMAX2*. Corresponding homologous protein sequences from 24 additional species were retrieved after the cloned sequence was submitted to the NCBI database for BLASTN alignment. All amino acid sequences from 25 species were aligned by using the CLUSTAL W method, and the alignment results were visualized using the ESPript 3.0 online protein sequence analysis tool. The results revealed that the MAX2 amino acid sequences of these 25 species contain a highly conserved F-box domain (Figure 1A). The sequence alignment results indicate that the F-box domain in the LpMAX2 protein may serve as a critical functional region involved in stress resistance in perennial ryegrass. The homologous MAX2 amino acid sequences from the 25 species were subjected to phylogenetic analysis using MEGA-X 10.2.6 software, with a neighbor-joining (NJ) tree constructed to assess the evolutionary relationships among these species. The results showed that the sequence from *Lolium perenne* shares a close phylogenetic relationship with other monocotyledonous Poaceae species, including *Lolium rigidum*, *Hordeum vulgare*, *Triticum aestivum*, *Oryza sativa*, *Zea mays*, *Setaria italic*, and *Sorghum bicolor*, and was more distant from dicotyledonous species (Figure 1B).

### 2.2. Subcellular Location of LpMAX2

Protein localization is a critical determinant of its biological function. To investigate the subcellular localization of LpMAX2, the *p1300-LpMAX2-EGFP* construct and *PJG188-LpMAX2-mCherry* were transiently transformed into *Nicotiana benthamiana* leaves via agrobacterium-mediated method. AtMAX2-mCherry (red fluorescence) co-localized with DAPI-stained nuclei, confirming its nuclear localization (Figure 1C). LpMAX2-EGFP (green fluorescence) similarly overlapped with DAPI signals (Figure 1D), demonstrating that LpMAX2 is a nuclear protein, analogous to *Arabidopsis* AtMAX2, suggesting functional conservation in their subcellular targeting mechanisms.

### 2.3. LpMAX2 Interacts with the SL Receptor D14

**Figure 2:**
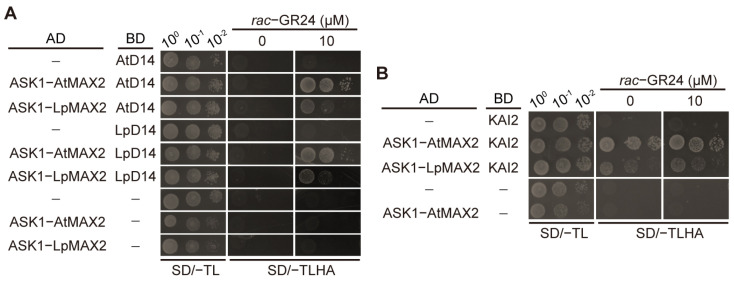
Interaction of LpMAX2 with D14 and KAI2. (**A**) Yeast two-hybrid assays demonstrating how LpMAX2 interacts with LpD14 and AtD14 in yeast strain AH109. Serial 10-fold dilutions of yeast cultures starting from OD600 1.0 were spotted onto a dropout medium lacking Leu, Trp, His and Ade with or without (Mock) 10 μM *rac*-GR24 after 5-day incubation. pGADT7 (AD) served as a negative control. (**B**) Yeast two-hybrid assays of LpMAX2-KAI2 interaction using the same system.

To elucidate the role of LpMAX2 in the SL signaling pathway, we performed yeast two-hybrid (Y2H) system. AD-ASK1-AtMAX2 and BD-AtD14 serve as positive controls. Although the interaction between LpMAX2-AtD14 was comparatively weaker than AtMAX2-AtD14, LpMAX2 can interact with both AtD14 (*Arabidopsis* D14) and LpD14 (perennial ryegrass D14) in an SL-dependent manner (Figure 2A). These results suggest that the interaction between LpMAX2 and D14 receptor proteins is evolutionarily conserved across species, and LpMAX2 likely plays a conserved functional role in the SL signal transduction pathway in perennial ryegrass, similar to its orthologs in other plants.

### 2.4. LpMAX2 Interacts with the Karrikin Receptor KAI2

Given that MAX2 proteins play crucial roles in both SLs and KAR signaling pathways, we further explored whether LpMAX2 is functionally involved in KAR signaling. AD-ASK1-LpMAX2 and BD-KAI2 were co-transformed into yeast cells, AD-ASK1-AtMAX2 and BD-KAI2 serve as positive control. The results demonstrate that LpMAX2 can physically interact with KAI2, a key receptor in the KAR signaling pathway. The LpMAX2-KAI2 interaction was weaker compared to the interaction between AtMAX2 and AtKAI2 (Figure 2B), indicating potential species-specific differences in binding affinity or structural compatibility.

### 2.5. Overexpression of LpMAX2 in Arabidopsis and Genotyping

**Figure 3:**
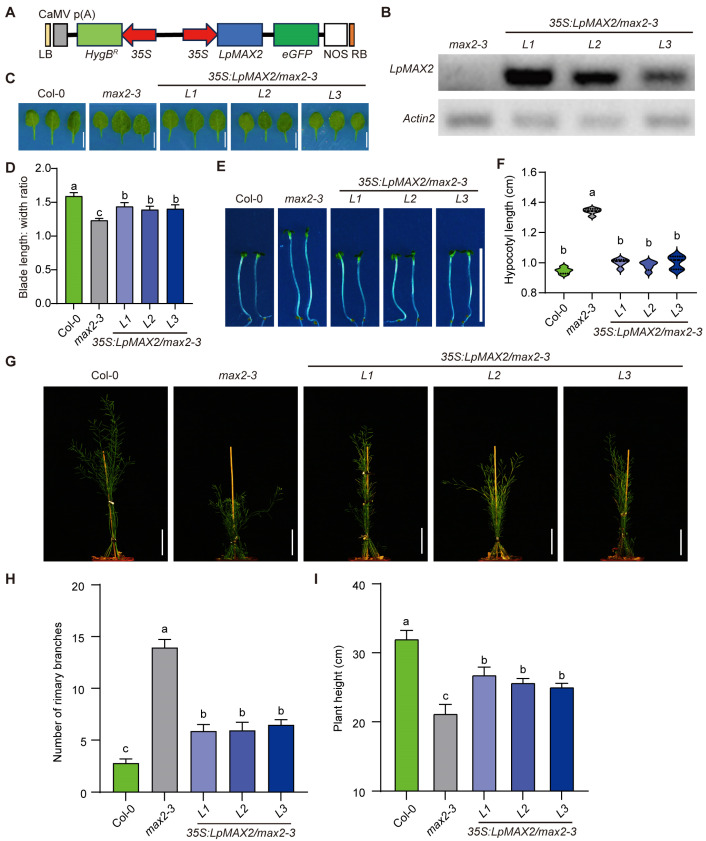
Phenotypes of *Arabidopsis* plants, including Col-0, *max2-3* and *LpMAX2* overexpression lines in *max2-3* background. The different lowercase letters indicated significant differences (*p* < 0.05). (**A**) A diagram showing *35S:LpMAX2* construct. 35S, cauliflower mosaic virus 35S promoter. NOS, nopaline synthase gene (NOS) terminator. RB, right border. LB, left border. HygB, *HygromycinB* gene. (**B**) Semi-quantitative RT PCR assays of *35S:LpMAX2* overexpression lines in *max2-3* background. (**C**) The 7th leaf of 18-day-old plants grown under LD photoperiod. Scale bar, 1 cm. (**D**) Leaf length, not including the petiole, and width in (**C**), *n* = 12. Data are means ± SD. (**E**) Phenotypes of 5-day-old seedlings were grown on half-strength Murashige and Skoog (MS) medium under continuous red light (~30 mmol µmol m^−2^ s^−1^). Bars, 10 mm. (**F**) Hypocotyl length of indicated genotypes shown in (**E**), *n* = 15. Data are means ± SD. (**G**) Shoot phenotypes of 45-day-old plants grown under LD photoperiod. Scale bar, 8 cm. (**H**) Primary rosette branch number of indicated genotypes shown in (**G**), *n* = 15. (**I**) Plant height of indicated genotypes shown in (**G**), *n* = 15. Data are means ± SD.

As a central regulatory component, MAX2 fulfills critical and non-substitutable functions across multiple aspects of plant development, including branching regulation, seed germination, root development, leaf morphology and hypocotyl elongation [20,29,30]. To investigate the role of LpMAX2 in plant growth and development, *LpMAX2* was overexpressed in *Arabidopsis thaliana* mutant *max2-3* via the *Agrobacterium*-mediated floral dip method. The obtained T1-generation *Arabidopsis* plants were screened with *hygromycinB*. A total of 21 positive lines were obtained and identified (Figure S1). The positive lines were identified via semi-quantitative RT-PCR with *LpMAX2* specific primers; the *Arabidopsis actin2* was used as a reference gene, and Col-0 and *max2-3* were used as negative controls. Six of them exhibited high expressions of *LpMAX2* (Figure 3B). We further cultivated these plants to harvest seeds, and five lines (*L1–L5*) successfully produced homozygous T3-generation seeds, which can be used for subsequent phenotypic analysis and functional validation.

### 2.6. LpMAX2 Partially Complements the Abnormal Phenotypes of Plant Development Regulated by SL/KAR Signaling

Three lines (*L1*–*L3*) of *35S:LpMAX2/max2-3*, Col-0 and *max2-3* grew in soil in the LD photoperiod for 18 days. The seventh leaf of the plants was measured, and the leaves of Col-0 were flatter and more elongated, while those of *max2-3* appeared curled, narrower, and shorter. In contrast, the leaves of three *35S:LpMAX2/max2-3* transgenic lines were more expanded and elongated compared to *max2-3* (Figure 3C). The leaf length-to-width ratio of *35S:LpMAX2/max2-3* transgenic lines were significantly greater than that of *max2-3* but smaller than that of Col-0 (Figure 3D). Branching and plant height of three transgenic lines (*L1–L3*) of *35S:LpMAX2/max2-3* and Col-0 and max2-3 were measured when plants were grown in soil for 42 days (Figure 3G). The number of primary branches of three *35S:LpMAX2/max2-3* transgenic lines was significantly reduced compared to *max2-3*, but this was significantly higher than that in Col-0. In terms of plant height, the three transgenic lines exhibited greater height than *max2-3*, but Col-0 was taller than the *35S:LpMAX2/max2-3* transgenic plants. The three transgenic lines showed varying degrees of phenotypic complementation in plant height (Figure 3H,I).

Furthermore, the hypocotyl elongation of Col-0, *max2-3* and three *35S:LpMAX2/max2-3* transgenic lines were measured after growing under 4 days of continuous red light (Figure 3E). The *max2-3* mutant exhibited the longest hypocotyl, while Col-0 had a shorter hypocotyl compared to *max2-3*, and three *35S:LpMAX2/max2-3* transgenic lines were significantly shorter than Col-0 but longer than *max2-3*. These results indicate that *LpMAX2* plays a conserved role in regulating leaf shape, hypocotyl elongation, primary branching, and plant height. However, LpMAX2 partially complements these phenotypes in the *max2-3* mutant.

### 2.7. Overexpression of LpMAX2 Completely Confers Drought Tolerance in Transgenic Arabidopsis

**Figure 4:**
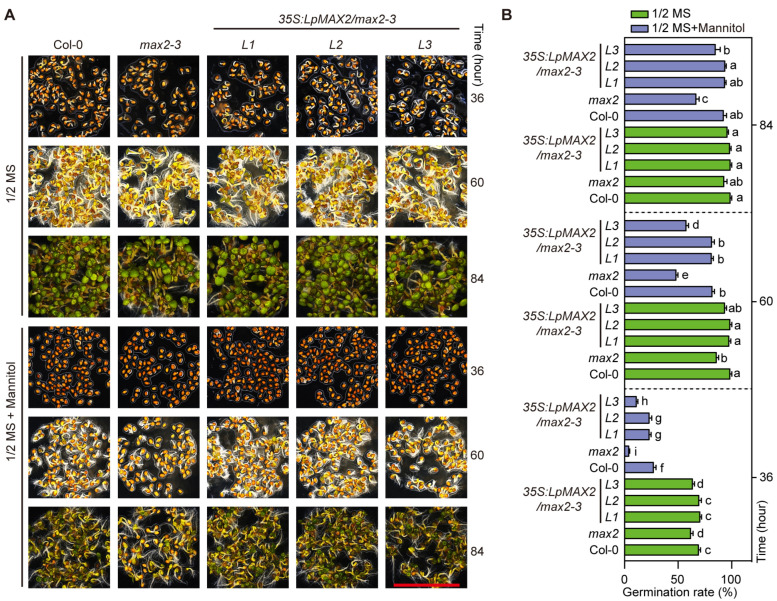
Germination analysis of Col-0, *max2-3* and *LpMAX2* overexpression lines in *max2-3* background under drought stress. The different lowercase letters indicated significant differences (*p* < 0.05). (**A**) Seed germination phenotypes on half-strength MS medium under LD photoperiod. Bars, 1 cm. (**B**) Quantitative germination rates under drought stress in (**A**).

Given that F-box proteins play a crucial role in plant responses to abiotic stresses such as drought, three *35S:LpMAX2/max2-3* transgenic lines alone with Col-0 and *max2-3* were subjected to drought stress at various growth phases. During the period of seed germination, *max2-3* exhibits a lower germination ratio than Col-0, and the ratio was more significant under drought treatment. Two *35S:LpMAX2/max2-3* transgenic lines (*L1* and *L2*) were similar to Col-0, while the other one (*L3*) exhibited a germination ratio that was lower than Col-0’s but higher than *max2-3*’s, both with and without drought stress (Figure 4A,B).

**Figure 5:**
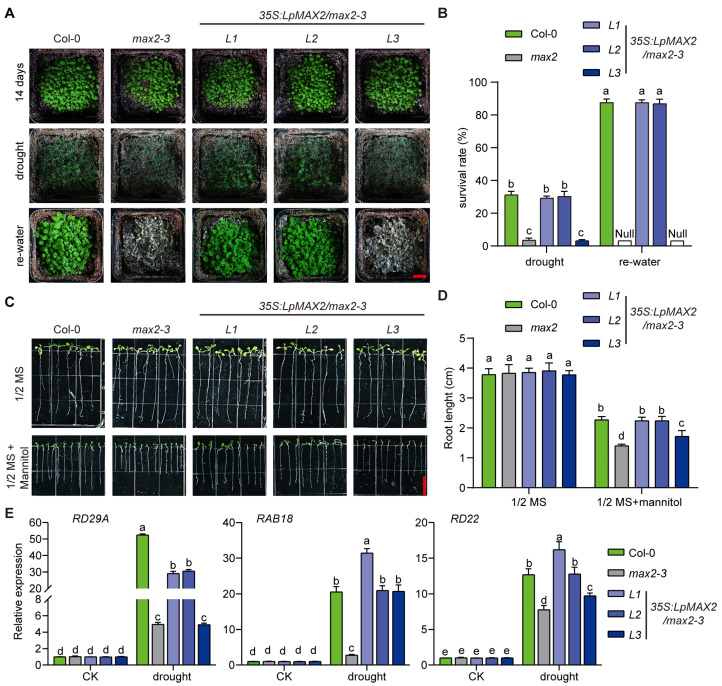
The phenotypes of *Arabidopsis* plants include Col-0, *max2-3* and *LpMAX2* overexpression lines in *max2-3* background under drought stress. The different lowercase letters indicate significant differences (*p* < 0.05). (**A**) Fourteen-day-old soil-grown seedlings under drought. Bars, 1 cm. (**B**) Survival rates of Col-0, *max2-3* and *LpMAX2* overexpression lines after drought and re-watering in (**A**), *n* = 15. Data are means ± SD. (**C**) Seven-day-old seedlings on half-strength MS medium under drought stress on LD photoperiod. Bars, 1 cm. (**D**) Root lengths of seedling in (**D**), *n* = 15. Data are means ± SD. (**E**) Expression pattern analysis of three drought-responsive genes after drought treatment. Data are means ± SD.

In addition, the root length of 7-day-old seedlings of Col-0, *max2-3* and three *35S:LpMAX2/max2-3* transgenic lines have no difference when grown on half-strength Murashige and Skoog (MS) medium; while *max2-3* was shorter than Col-0 under drought stress (1/2 MS + mannitol), two *35S:LpMAX2/max2-3* transgenic lines (*L1* and *L2*) were similar to Col-0 and the other one (*L3*) exhibited a length that was shorter than Col-0 but higher than *max2-3* (Figure 5C,D). Moreover, for 14-day-old seedlings of Col-0, *max2-3* and three *35S:LpMAX2/max2-3* transgenic lines grown on soil, drought conditions were imposed by withholding irrigation for 11 days, then re-watering for 5 days (Figure 5A). Under drought conditions, the survival rate of Col-0 was significantly higher than *max2-3*; two *35S:LpMAX2/max2-3* transgenic lines (*L1* and *L2*) were similar to Col-0 and the other one (*L3*) was similar to *max2-3*. After re-watering, the survival rate of Col-0 was significantly higher than it was under drought conditions; two *35S:LpMAX2/max2-3* transgenic lines (*L1* and *L2*) were similar to Col-0, but *max2-3* and *35S:LpMAX2/max2-3-L3* were barely alive (Figure 5B). And the water loss of the rosetta leaves of Col-0 and two *35S:LpMAX2/max2-3* transgenic lines (*L1* and *L2*) was slower than in *max2-3* and *35S:LpMAX2/max2-3-L3* (Figure S2).

To elucidate *LpMAX2*-mediated drought stress responses, we quantified the expression of three well-characterized drought-inducible marker genes (*RD29A*, *RAB18*, and *RD22*) in Col-0 [31,32], *max2-3* and three *35S:LpMAX2/max2-3* transgenic lines. While no genotype-specific variations were detected in unstressed plants, drought stress elicited line-dependent activation profiles of the drought-inducible genes *RD29A*, *RAB18*, and *RD22*. A significant reduction in stress induction was observed for all three genes in *max2-3*, and the *35S:LpMAX2/max2-3-L3* complementation lines showed similarly reduced *RD29A* induction. The other two *35S:LpMAX2/max2-3-L1/L2* complementation lines displayed normal drought induction kinetics of all three marker genes, achieving complete phenotypic rescue comparable to Col-0. These results indicate that *LpMAX2* can rescue the drought-sensitive phenotype of *max2-3* mutants, and it may be attributed to *LpMAX2* enhancing the expression of drought-responsive genes.

## 3. Discussion

As a high-value perennial graminoid species cultivated extensively for both forage production and turf applications, perennial ryegrass (*Lolium perenne* L.) exhibits tillering behavior and vertical growth dynamics that constitute critical agronomic traits requiring further mechanistic investigation [25,33,34]. F-box protein MAX2 is a key strigolactone/karrikin signaling component that regulates diverse biological processes, including plant architecture (branching and height), photomorphogenesis, stress response and senescence [30].

This study isolated and characterized a MAX2 homolog, designated *LpMAX2* from perennial ryegrass (*Lolium perenne* L.). The *LpMAX2* encodes a protein containing highly conserved F-box domain (Figure 1A). Subcellular localization confirmed that both LpMAX2 and AtMAX2 localize in the nucleus, consistent with previous studies [30]. LpMAX2 was shown to interact with both AtD14 and LpD14 in an SL-dependent manner, and interact with KAI2 (Figure 2A,B), suggesting its conserved role in SL/KAR signaling in perennial ryegrass.

Comparative analyses of MAX2 orthologs (GhMAX2 in cotton, MsMAX2 in *Medicago sativa*, ShMAX2 in *Striga asiatica*, OaMAX2 in *Orobanche aegyptiaca*, and PvMAX2 in *Panicum virgatum*) have revealed conserved roles in regulating both plant architecture [35,36,37,38] and drought stress responses [22,23,24]. For example, overexpression of *OaMAX2* from *Orobanche aegyptiaca* completely restored most phenotype defects in both development and drought responses [37]. However, in our study, LpMAX2 only partially complemented *max2-3* mutant development phenotypes, including leaf morphology, hypocotyl elongation, branching and plant height. Conversely, LpMAX2 completely restored the drought tolerance of the *max2-3* mutant. Through qRT-PCR analysis, the expression patterns of drought-responsive marker genes (*RD22*, *RD29A*, and *RAB18*) were detected in Col-0, *max2-3* and *LpMAX2* overexpression lines in *max2-3* background under drought treatments. The observed gene expression profiles and drought-induced phenotypic changes were consistent with those reported in other species. All these findings also confirmed the evolutionary conservation roles of LpMAX2 in SL/KAR signaling-mediated developmental regulation. Additionally, in some studies, ectopic expression of grass MAX2s also failed to completely complement all phenotypes, especially development phenotypes [36]. However, in these studies, nearly all ectopic expressions of these MAX2s fully rescue the ability to resist drought and saline stresses.

The partial complementation of phenotypes such as branching and hypocotyl elongation by LpMAX2, but not full complementation, may likely result from weaker interactions between LpMAX2 and AtD14/AtKAI2 (Figure 2), leading to reduced transduction of SL/KAR signaling. Previous research showed that the branching and hypocotyl elongation phenotypes of the *Arabidopsis max2-3* mutant were partially rescued by overexpression of *Orobanche aegyptiaca OaMAX2* [37], whereas overexpression of *SsMAX2* from *Sapium sebiferum* suppressed both shoot branching and hypocotyl elongation significantly more than wild-type plants [22]. This discrepancy in phenotypic complementation likely reflects evolutionary divergence in the MAX2 proteins among different species. However, LpMAX2 fully rescues the drought-resistant phenotype associated with SL/KAR signaling, suggesting that even weak SL/KAR signaling can strongly influence drought stress responses. This observation implies that SL/KAR-mediated regulation of drought stress resistance is an evolutionarily conserved mechanism.

Overall, in this study, an F-box protein LpMAX2 was identified from perennial ryegrass (*Lolium perenne* L.). *LpMAX2* retains evolutionarily conserved functions in regulating key developmental processes and drought resistance. Moreover, increased drought resistance observed in the *smax1/2* and *smxl6/7/8* mutants, which are implicated in strigolactone and karrikin signaling [39,40,41,42], suggests that targeting their homologous pathways in perennial ryegrass could lead to the development of drought-resistant cultivars. This study significantly advances our understanding of the functional conservation of MAX2 proteins and provides a promising molecular target for improving drought tolerance in perennial ryegrass (*Lolium perenne* L.), which will accelerate the development of drought-resistant cultivars and facilitate their cultivation in arid and semi-arid regions.

## 4. Materials and Methods

### 4.1. Plant Materials and Growth Conditions

The *Arabidopsis thaliana max2-3* mutant (SALK_092836) was in the Col-0 background. Seeds were surface-sterilized with 20% (*v*/*v*) bleach for 10 min, followed by six rinses with sterilized ultrapure water. After sterilization, the seeds were sown on half-strength Murashige and Skoog (1/2 MS) medium supplemented with 0.8% (*w*/*v*) agar and vernalized at 4 °C for 3 days. Plants were grown in a controlled-environment chamber at 22 °C under a 16 h light (100 μE m^−2^ s^−1^)/8 h dark photoperiod (LD photoperiod).

Perennial ryegrass cultivar “flash” was bought from Zhengdao Seed Industry Co., Ltd. (Beijing, China). “Flash” seeds were germinated and grown in sandy loam soil in a growth chamber at 20/15 °C (day/night) under a 16 h light (100 μE m^−2^ s^−1^)/8 h dark photoperiod (LD photoperiod).

### 4.2. Gene Isolation and Bioinformatic Analysis

Total RNA was isolated using TRIzol reagent (Invitrogen, Carlsbad, CA, USA) following the manufacturer’s protocol. First-strand cDNA was synthesized from 1 μg of total RNA using the PrimeScript™ RT Reagent Kit with gDNA Eraser (TaKaRa, Shiga, Japan) to eliminate genomic DNA contamination. *LpMAX2*-specific primers were designed using Primer 6.0 for PCR amplification. Multiple sequence alignment was performed using ClustalW 2.0, and a phylogenetic tree was constructed in MEGA 6.0.6 using the neighbor-joining method with 1000 bootstrap replicates [43]. All primers used for genotyping and vector construction are listed in Supplementary Table S1.

### 4.3. Subcellular Localization of MAX2 Proteins

To analyze the subcellular localization of AtMAX2 and LpMAX2, the coding sequences (CDS) of *AtMAX2* and *LpMAX2* without stop codons were amplified and cloned into *PJG188-mCherry* and *p1300-eGFP*, respectively, to generate *35S:AtMAX2-mCherry* and *35S:LpMAX2-eGFP* constructs. All constructs were individually transformed into the *Agrobacterium tumefaciens* GV3101 strain via the freeze–thaw method. Positive transformants were cultured in a LB medium with appropriate antibiotics at 28 °C for 16 h, harvested by centrifugation, and resuspended in infiltration buffer (10 mM MES, pH 5.6, 10 mM MgCl_2_, and 150 μM acetosyringone). Bacterial suspensions containing different constructs were mixed in equal proportions to achieve a final OD600 of 0.5 for each strain. The positive transformants were co-infiltrated with P19 strain into 4-week-old *N. benthamiana* leaves. After infiltration, plants were maintained under 16 h dark/8 h light cycles for 60–72 h. Fluorescence was detected using a confocal laser scanning microscope (Leica Microsystems Inc., Buffalo Grove, IL, USA). Nuclei were counterstained with 4′,6-diamidino-2-phenylindole (DAPI, MA, Thermo Fisher, USA) to facilitate cellular compartment identification. Fluorescence signals were captured using appropriate filter sets: 488 nm excitation/505–530 nm emission for eGFP, 587 nm excitation/610 nm emission for mCherry, and 358 nm excitation/461 nm emission for DAPI.

### 4.4. Yeast Two-Hybrid Assays

The CDS of *LpD14*, *AtD14* and *KAI2* were cloned into yeast expression vectors pGBKT7, while *ASK1* (AT1G10940) fused with the N terminus of *LpMAX2* or *AtMAX2* was cloned into pGADT7 to generate pGADT7-ASK1-LpMAX2 and pGADT7-ASK1-AtMAX2 constructs. Yeast two-hybrid assays were performed as previously described [44]. Bait and prey constructs were co-transformed into the yeast strain AH109 using the lithium acetate-mediated method. All transformants were selected on synthetic defined (SD) medium (SD/-Leu/-Trp double dropout media) plates for 3–5 days. Positive transformants were cultured overnight in SD/-Leu/-Trp liquid media; cultures were normalized to OD600 = 1.0, serially diluted (10-fold) to final concentrations of OD600 = 1.0, 0.1 and 0.01, then spotted onto selective growth medium (SD/-Leu/-Trp/-His) plates containing 10 μM *rac*-GR24 or DMSO (solvent control, MA, Thermo Fisher, USA). Plates were incubated at 30 °C for 3 to 5 days to assess interactions.

### 4.5. Generation and Genotyping of LpMAX2 Overexpression Transgenic Lines

For overexpression lines, the CDS of *LpMAX2* was cloned into pCAMBIA1300-eGFP vector to generate the *35S:LpMAX2-eGFP* construct, which was stably transformed into the *Arabidopsis max2-3* background using the *Agrobacterium*-mediated floral dip method. Transformed plants were screened with 10 mg hygromycin B until homozygous lines were obtained.

Positive transgenic lines were verified by semi-quantitative RT-PCR. Total RNA was extracted from positive lines, Col-0 and *max2-3* plants, and cDNA was synthesized as described above. *LpMAX2* was amplified using the *LpMAX2*-specific primers in 30-cycle, with *AtActin2* (At3g18780) serving as an internal control.

### 4.6. Seedling Photomorphogenesis Assay

Seedling photomorphogenesis assay was conducted according to Soundappan [17]. Seeds were treated with 3 h of white light (~50 to 70 μmol m^−2^ s^−1^) at 21 °C, followed by 21 h of darkness at 21 °C, then grown for 4 days at 21 °C under continuous red light (~30 mmol μmol m^−2^ s^−1^). Hypocotyl lengths were measured from digital images using ImageJ software (fiji is just ImageJ).

### 4.7. Drought Stress Treatments

For plant drought stress treatment in soil, plants were grown in a controlled-environment chamber at 22 °C under a 16 h light (100 μE m^−2^ s^−1^)/8 h dark photoperiod for 14 days to establish uniform growth prior to completing the withholding of irrigation for 11 days, followed by re-watering to assess recovery.

For plant drought stress treatment in half-strength Murashige and Skoog (MS) medium, seeds of Col-0, *max2-3* and *LpMAX2* overexpression lines were sown on 1/2 MS containing 0.8% (*w*/*v*) agar as a control group and were sown on 1/2 MS containing 0.8% (*w*/*v*) agar with 300 mM mannitol (MA, Thermo Fisher, USA) as an experimental group, then vernalized at 4 °C for 3 days. Plants were grown in a controlled-environment chamber at 22 °C under a 16 h light (100 μE m^−2^ s^−1^)/8 h dark photoperiod. Germination rates were analyzed at 36, 60 and 84 h, and the primary root lengths were measured on the 7th day post-germination using Image (fiji is just ImageJ) analysis software.

### 4.8. RNA Extraction and RT-qPCR

Total RNA was isolated from transgenic positive lines, Col-0 and *max2-3* plants, and cDNA libraries were synthesized as described above. RT-qPCR was performed using SYBR Green Supermix (TransGen Biotech Ltd., Beijing, China) on a QuantStudio 1 Real-Time PCR System (Applied Biosystems, Foster City, CA, USA). *Actin2* served as the reference gene, and relative gene expression levels were calculated using the 2-ΔΔCt method. Three independent biological replicates were analyzed for each gene. All primer sequences are provided in Supplementary Table S1.

### 4.9. Statistical Analysis

All data were derived from three independent replicates of each experiment. Statistical analysis was performed with one-way ANOVA, followed by Tukey’s test method using SPSS 22.0 software, with results presented as mean ± standard deviation. Statistical significance was marked with asterisk for *p*-values: *p* < 0.05 (*), *p* < 0.01 (**) and *p* < 0.001 (***).

## 5. Conclusions

Collectively, this study demonstrates the conserved roles of LpMAX2 in SL/KAR signaling pathways, highlighting its particularly critical function in drought adaptation in perennial ryegrass. This study identifies a promising genetic target for enhancing stress resilience in forage grasses through biotechnological approaches.

## Data Availability

The original contributions presented in this study are included in the article/Supplementary Material. Further inquiries can be directed to the corresponding author(s).

## Supplementary Materials

The following supporting information can be downloaded at https://www.mdpi.com/article/10.3390/ijms27010031/s1.
