# Supplementary material for: LpMAX2 Is a Strigolactone/Karrikin Signaling Component in Perennial Ryegrass (Lolium perenne L.)
Source: Int J Mol Sci. 2025 Dec 19;27(1):31. doi: 10.3390/ijms27010031 (PMC12785265; doi:10.3390/ijms27010031)
Supplement: Supplementary file 1 [file ijms-27-00031-s001.zip › LpMAX2 Figures S1 and S2.pdf]

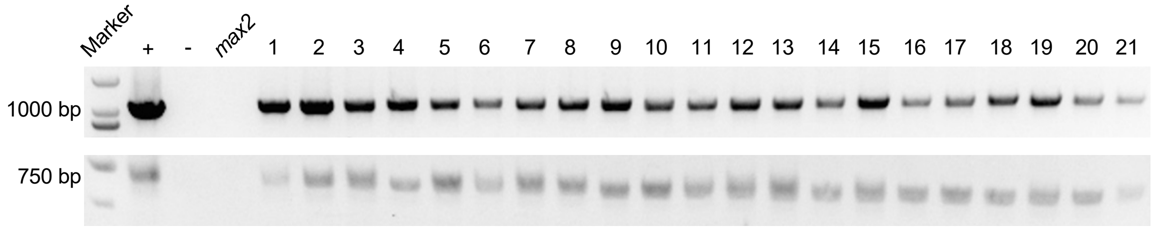

Figure S1. Identify positive lines via PCR. Up, *LpMAX2* specific primers were used. Down, *hygromycin B* specific primers were used.

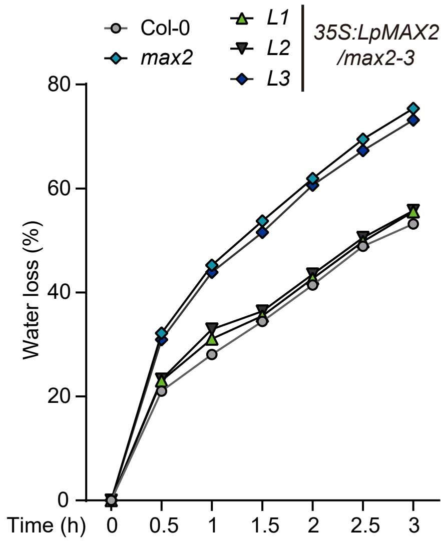

Figure S2. Water loss of rosetta leaves of Col-0, *max2-3* and *LpMAX2* overexpression lines after detached in 25°C, n=15, Data are means ± SD.
